# Treatment of primary epididymal adenocarcinoma: a case report and review of the literature

**DOI:** 10.1186/s13256-024-04590-4

**Published:** 2024-06-10

**Authors:** Jianhua Xiao, Yan You, Ziqiang Dong, Qi Wu, Honggang Yuan

**Affiliations:** 1 Department of Urology, The First Clinical Medical College of China Three Gorges University, Yichang Central People’s Hospital, Yichang, China; 2 Department of Pharmacy, The Second Clinical Medical College of China Three Gorges University, Three Gorges University Renhe Hospital, Yichang, China; 3 Department of Pathology, The First Clinical Medical College of China Three Gorges University, Yichang Central People’s Hospital, Yichang, China

**Keywords:** Primary epididymal malignant tumors, Diagnosis, Treatment surgery, Case report

## Abstract

**Background:**

Epididymal tumors, especially malignant tumors, have low incidence and are rare in our clinical work. However, they may progress quickly and have poor prognosis. For such rare clinical cases with extremely low incidence rates, and as they are prone to misdiagnosis and missed diagnosis and have a very poor prognosis, clinical workers need to pay special attention and consider the possibility of primary epididymal malignant tumors.

**Case report:**

A 63-year-old Chinese male patient from Asia was admitted due to scrotal pain. Upon examination, an abnormal lesion was found in the right epididymal region. After thorough evaluation, surgical resection was performed, and the postoperative pathological result confirmed the presence of epididymal adenocarcinoma. After further ruling out secondary lesions, primary epididymal adenocarcinoma was considered. Right retroperitoneal lymph node dissection was performed under laparoscopic for treatment, and 1/11 lymph node metastasis was detected after surgery. The patient is currently under close follow-up.

**Conclusions:**

The number of clinical cases of primary epididymal malignant tumors is very limited, there is currently no standardized diagnosis and treatment process, and there is a lack of systematic evaluation methods regarding the effectiveness of different treatment options such as chemotherapy, radiotherapy, immunotherapy, and targeted therapy. In addition, the outcome is difficult to predict. In this article, we reviewed relevant literature and systematically elaborated on the diagnosis and treatment of epididymal malignant tumors, hoping to provide useful information for relevant experts.

## Background

Primary malignant tumors of the epididymis are rare clinical diseases, and the incidence rate is less than 7% of overall male genital system tumors. The age range of its onset is wide, but it is more common between the ages of 30 years and 50 years, which is 10 years later than the average age of onset for benign tumors of the epididymis. Among them, the age distribution of patients with different pathological types varies, and embryonic rhabdomyosarcoma occurs earlier. To date, the youngest patient reported in China is only 14 months old. The onset age of adenocarcinoma is relatively older, generally over 40 years [1]. There is no relevant report on the difference in incidence rate between different regions or races. Primary malignant tumors of the epididymis are mostly unilateral, with more tumors on the left side. Moreover, tumors are mostly located in the tail of the epididymis, and bilateral cases are very rare. Due to their anatomical characteristics, epididymal tumors are theoretically easy to find. However, as the hidden clinical symptoms and the fact that epididymal masses are mostly benign tumors, chronic inflammatory nodules, or sperm stasis, the incidence of malignant tumors is low, leading to low alertness of clinical physicians to them, thus delaying the time of diagnosis and treatment. At the same time, epididymal malignant tumors are highly malignant tumors and prone to metastasis early with poor prognosis. Moreover, most of the domestic and foreign literature consists of case reports rather than systematic research. Therefore, a relatively standardized diagnosis and treatment mode has not yet been determined [2].

## Case report

**Fig. 1:**
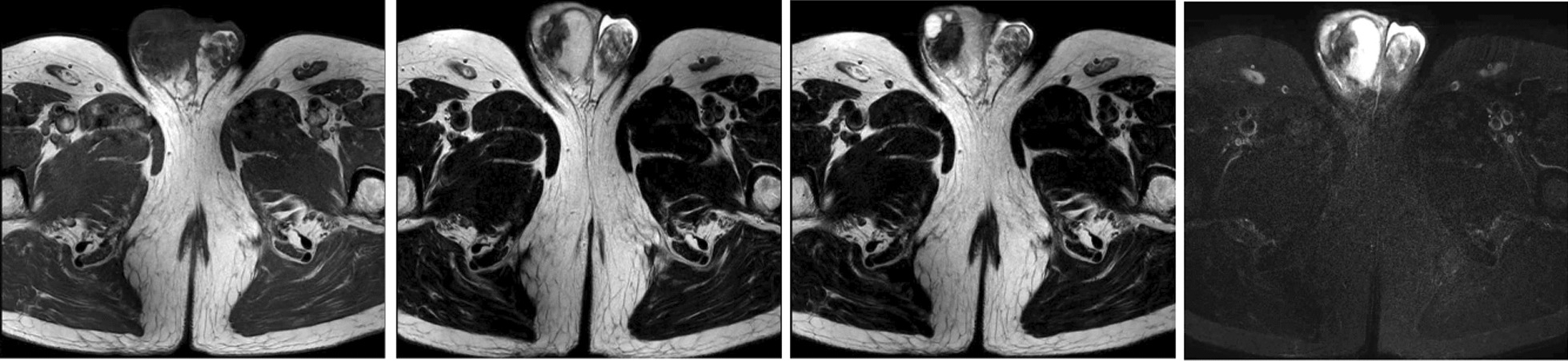
Preoperative magnetic resonance imaging (August 2021) showing mixed signals of cystic and solid nature in the right epididymis

A 63-year-old Chinese male patient from Asia visited our hospital on 1 August 2021, due to “swelling and pain in the right testicle for over a year” who had a history of surgical treatment for left testicular hydrocele and was without other special medical experiences and allergy history regarding food and drugs, no history of exposure to significant carcinogenic factors, and no obvious family history of tumors. Physical examination: normal vital signs, height 178 cm, weight 74 kg, no swelling of superficial lymph nodes throughout the body, intact scrotal skin, no redness, swelling or pain. The size and morphology of the left testicular epididymis were normal, with slightly lower activity. The size of the right testicle was close to normal, and there was a nodule about 4 cm × 4 cm in size in the upper and posterior epididymal area. The texture was hard, with poor mobility and no obvious squeezing pain. Scrotal ultrasound showed an irregular cystic mass in the right epididymis area that was tightly adhered to the right testicle, exhibiting an uneven mixed echo mass with unclear boundaries. The size of the lump was approximately 4 cm × 3 cm × 2 cm. Magnetic resonance imaging (MRI) prompted the following description: right epididymis was full; within it, there was a range of approximately 2.6 cm × 2.8 cm × 2.1 cm mixed signals of cystic and solid nature; boundary was not smooth, especially in the junction area to the upper edge of the right testicle (Fig. 1). Retroperitoneal computed tomography (CT) and chest CT did not show important positive results. The levels of alpha fetoprotein (3.6 ng/mL), human chorionic gonadotropin (0.3 mIU/mL), and lactate dehydrogenase (220 IU/L) were all within the normal range.

### Preliminary clinical diagnosis

#### Tumor of the right epididymis

**Fig. 2:**
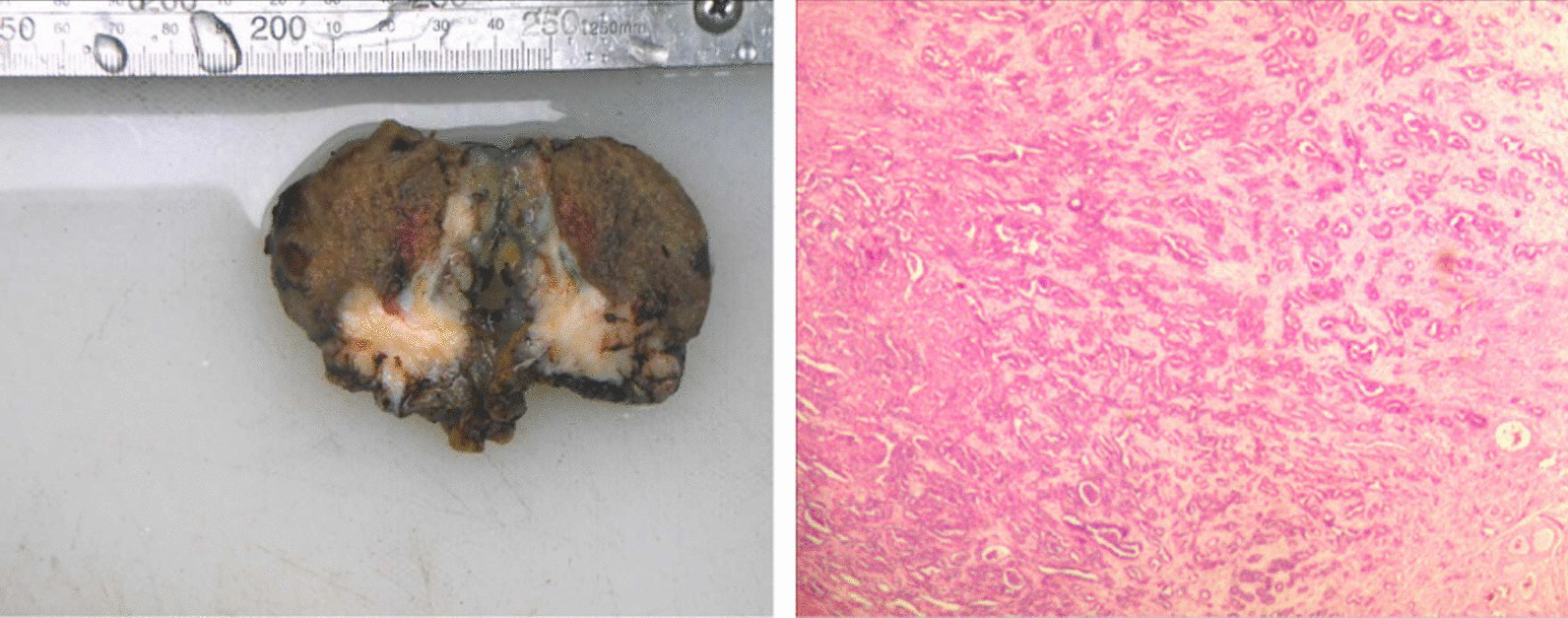
Macroscopic and electron microscopic photos of postoperative specimens, Hematoxylin and eosin (HE) ×40

The nature of the patient’s right epididymis tumor was uncertain, and a malignant tumor could not be ruled out. After sufficient communication with the patient and his family on feasible management measures, the patient chose to undergo radical resection of the right testicular epididymis directly. With sufficient preparation, we performed radical resection of the right testis and epididymis through a right inguinal incision. The postoperative gross specimen was described as follows: the right testicle with the epididymis was completely cut, measuring 5.5 cm × 5 cm × 3 cm in size; the epididymis was 3 cm × 2.5 cm × 2 cm in size; upon incision, gray yellow spongy testicular tissue was observed, and a gray white lump with a size of approximately 3.5 cm × 3 cm × 2 cm was visible in the epididymis; the surface of the lump was gray white and solid in nature; under the microscope, the tumor cells had varying structures, large nuclei, obvious atypia, deeply stained cytoplasm, and glandular-like structures (Fig. 2).

The immunohistochemical results were as follows: PCK(+), EMA(+), CEA(−), WT-1(+), CK5/6(+), CR(calretinin)(+), CD30(−), SALL4(−), Ki-67(20%), OCT-3/4(−), CK7(+), β-catenin(cytoplasm)(+), inhibin-α(−), MC(HBME-1)(−), D2-40(−), pax-8(−), and Cam5.2(+). Microscopic morphology and immunohistochemical results supported epididymal adenocarcinoma with testicular invasion. Perineural invasion was identified, and no tumor thrombus was found in the vessels. Further gastroscopy, colonoscopy, electronic laryngoscopy, neck enhanced MRI, and positron emission tomography (PET)-CT examinations did not indicate the possibility of tumors in other parts of the body. Therefore, we considered the final diagnosis as primary adenocarcinoma of the right epididymis. Considering the high malignancy of primary adenocarcinoma of the epididymis and its susceptibility to retroperitoneal lymph node metastasis, some scholars have pointed out that retroperitoneal lymph node dissection should be performed not only in cases with lymph node enlargement, but also as a preventive treatment for clinical N0 patients [3]. Therefore, retroperitoneal lymph node dissection was recommended for this patient.

**Fig. 3:**
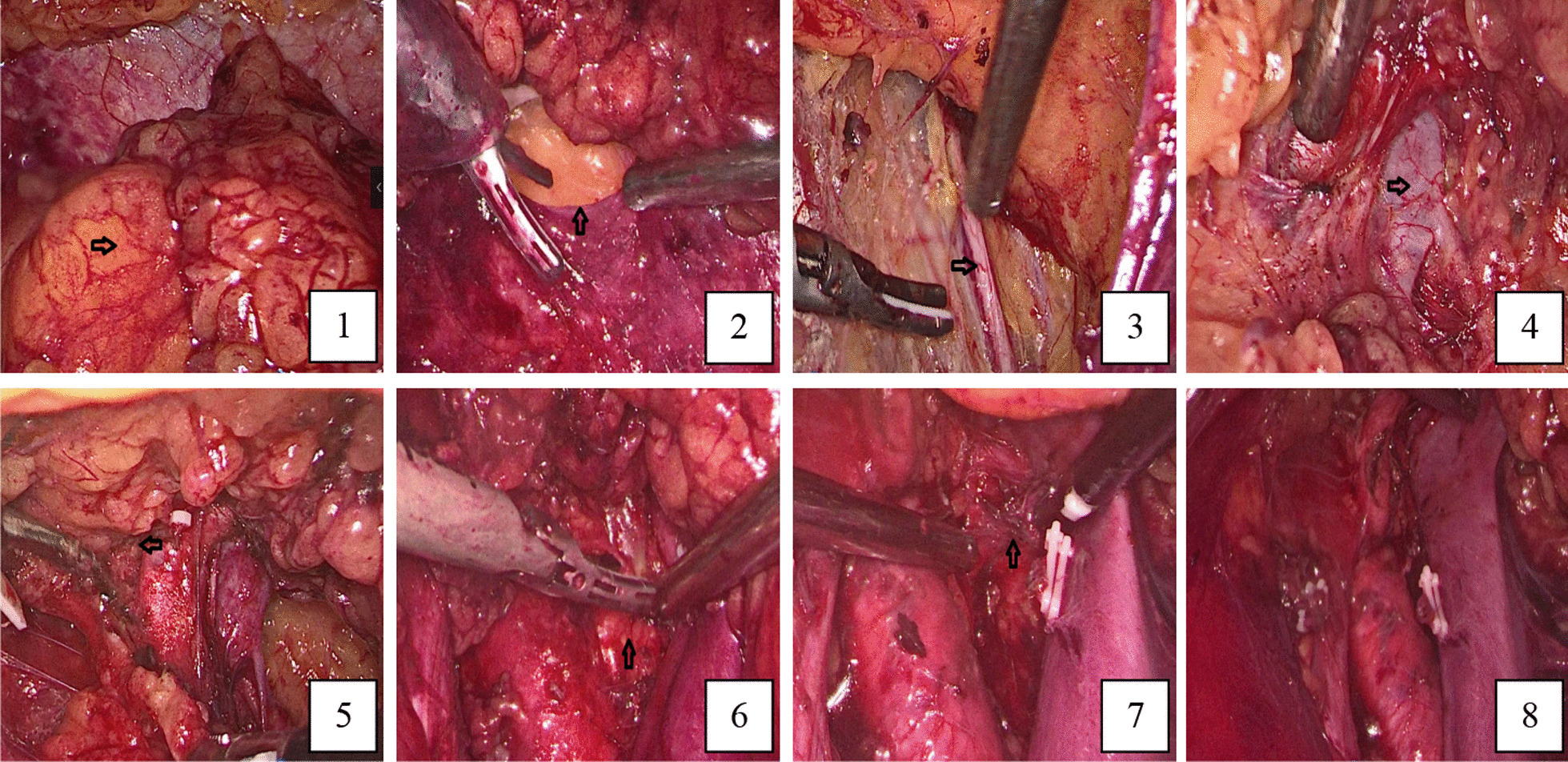
Screenshots of the right retroperitoneal lymph node dissection under laparoscopy: (1) clearing retroperitoneal fat; (2) opening perirenal fascia (3) exposing the ureter; (4) exposing vena cava (5) the upper edge of the dissection exceeded the level of the renal artery; (6) exposing the aorta and lymph tissues between the abdominal aorta and vena cava; (7) exposing the contralateral renal vein; (8) overall view after dissection. The black arrows indicate the main anatomical structures described in each image

The laparoscopic retroperitoneal lymph node dissection was performed 2 weeks later. During the surgery, we removed the entire right retroperitoneal lymphoid adipose tissue and checked the wound surface to ensure that there was no obvious bleeding point. After repeatedly rinsing the wound with sterile distilled water, we removed the specimen tissues (Fig. 3). The overall appearance of the postoperative specimens was described as follows: a piece of gray yellow adipose tissue with a size of 7.5 cm × 4.5 cm × 2.5 cm; several nodules with a diameter of 0.5–3 cm were observed inside, and all nodules were taken; The microscopic examination results were as follows: 11 lymph nodes were found, with one lymph node showing cancer metastasis (1/11).

### Postoperative diagnoses

- Primary malignant tumor of the right epididymis,
- Adenocarcinoma,
- Secondary malignant tumor in the right retroperitoneal lymph node.

### Follow-up situation

After multidisciplinary discussions and evaluations, the surgery completely removed the tumor tissue. Under sufficient communication with the patient and their family on feasible treatment measures, they decided not to accept adjuvant measures such as radiotherapy and chemotherapy, and strict follow-up had been chosen. At 3 months, 6 months, 9 months, 12 months, 18 months, 24 months, and 30 months after surgery, the imaging showed no progression. As of now, no additional adjuvant treatment has been added, and close follow-up is continuing.

## Discussion

Primary malignant tumors of the epididymis are rare clinical diseases, there is currently no standardized diagnosis and treatment process, and there is a lack of systematic evaluation methods of the effectiveness regarding different treatment options such as chemotherapy, radiotherapy, immunotherapy, and targeted therapy. We want to review relevant literature and systematically elaborate on the diagnosis and treatment of epididymal malignant tumors in this paper.

### Risk factors of primary malignant tumors

The existing relevant literature has not systematically elucidated the high-risk factors for primary epididymal malignant tumors, but there are currently no relevant reports on whether cryptorchidism or testicular insufficiency is related to the occurrence of epididymal malignant tumors. However, some literature has mentioned the relationship between cryptorchidism or undescended testis and epididymal malignant tumors, but whether there is a relationship between them is still uncertain [4].

### Clinical symptoms

Primary malignant tumors of the epididymis lack specific symptoms and signs, mostly manifesting as painless masses within the scrotum, mostly located in the tail of the epididymis, followed by the head. Some patients have local soreness or pain accompanied by a sense of heaving. The masses often exhibit progressive growth, with a diameter often greater than 3 cm and unclear boundaries with surrounding tissues. They can be accompanied by thickening of the affected spermatic cord or vas deferens, appearing nodular or beaded on palpation. When accompanied by hydrocele of the tunica vaginalis, the testicular volume substantially increases, and the transparency test can be positive. Some patients also have acute epididymitis, and the affected testis and epididymis are also considerably enlarged, with obvious tenderness, redness, and swelling of the scrotal skin and a notable increase in skin temperature. In severe cases, purulent secretions may be present [5]. When tumors metastasize locally or remotely, corresponding symptoms and signs are observed.

### Laboratory examinations

Early clinical laboratory examinations may show no important abnormalities, and in cases of acute inflammation, white blood cell count, neutrophil count, C-reactive protein, procalcitonin, and other indicators in the blood can be considerably higher than the normal range. When the disease progresses to the terminal stage, important abnormalities in various blood indicators may occur, such as electrolyte disorders, severe hypoproteinemia, liver and kidney dysfunction, and elevated alkaline phosphatase. As of now, there are no reports suggesting specific tumor markers for primary epididymal malignant tumors. Common tumor markers related to testicular malignant tumors, such as blood alpha fetoprotein (AFP), lactate dehydrogenase (LDH), human chorionic gonadotropin (HCG), and carcinoembryonic antigen (CEA), are mostly normal. However, secondary malignant tumors in the epididymis may exhibit specific abnormal tumor markers, such as a substantial increase in blood prostate specific antigen (PSA) when prostate malignant tumors metastasize to the epididymis [4].

### Imaging examinations

Malignant tumors of the epididymis have a complex tissue source and thus lack typical imaging manifestations. High-resolution ultrasound is considered the preferred imaging examination method for testicular and epididymal tumors due to its noninvasive and convenient advantages. It can provide some common features: irregular and large (usually larger than 3 cm) cystic, mixed cystic and solid, or solid masses in the epididymal or epididymal spermatic cord area. Tumors are prone to invading surrounding tissues such as the spermatic cord, testicles, or scrotal skin. The morphology of the testes and epididymis is often unclear when the tumor is large. Epididymal malignant tumors often have an abundant blood supply, but the manifestations of tumors from different tissue sources vary. They can be accompanied by testicular hydrocele and considerable thickening of the spermatic cord. At the same time, ultrasound can also be used to carefully explore the bilateral spermatic cord area, pelvic cavity, and retroperitoneal area to determine whether there is lymph node enlargement [6]. CT can also clearly display abnormal density lesions near the testicles within the scrotum and clarify the relationship between the tumor and the epididymis, and enhanced scanning can assist in determining the blood supply of the tumor. MRI often shows irregular nodules on the lateral side of the testis, closely related to the epididymis. The internal signals of the nodules are uneven, and the enhanced scan can show gradual enhancement, mainly with edge enhancement. Magnetic resonance imaging diffusion weighted imaging (MRI-DWI) is significantly limited in diffusion, and MRI can also be useful in determining local and distant metastasis. Craniocerebral CT or MRI, emission computed tomography (ECT) bone scan, positron emission computed tomography (PET-CT), among others, can all be used as supplementary examinations [7].

### Methods of tumor tissue acquisition for pathological diagnosis

Due to its rarity and lack of specific clinical manifestations, it is difficult to make a clear diagnosis before surgery for primary malignant tumors of the epididymis. Fu *et al*. [8, 9] reported that there were very few cases of epididymal tumors that were clearly diagnosed before surgery, and most of them were misdiagnosed as other epididymal diseases, with a misdiagnosis rate of 63%. Regarding preoperative needle aspiration cytology examination, Smith *et al*. reported that the examination could lead to approximately 0.006% malignant tumor implantation, while Andersson *et al*. [10, 11] concluded that the possibility of implantation metastasis through fine needle aspiration was basically negligible, confirming the feasibility of this method. However, after careful analysis of the relevant literature, it was found that needle aspiration cytology is not a routine choice for patients with relatively limited lesions. More people choose to undergo direct surgical exploration followed by intraoperative rapid freezing examination, the results of which guide further comprehensive treatment measures.

### Pathological types of malignant tumors in the epididymis

Epithelial malignant tumors of the epididymis are relatively rare, and most of them originate from interstitial tissue. Among all pathological types, adenocarcinoma is more common in clinical practice, followed by embryonic rhabdomyosarcoma [12]. The remaining tissue types are relatively complex, and different pathological types have different clinical characteristics and prognosis outcomes, which are often difficult to determine before surgery and postoperative pathological analysis. The main types of primary epididymal malignant tumors that are commonly reported in domestic and foreign literature are presented in Table 1.

**Table 1:** The main pathological types of primary epididymal malignant tumors

Tumor category	Histogenesis	Macroscopic view of tumor tissues	Histological characteristics and microscopic observation	Common immunohistochemical indicators	Common imaging features	Common transfer situations	Main treatment methods
Adenocarcinoma [3, 13–15]	Epididymal glandular epithelium	White or yellowish brown, with a hard texture and often accompanied by necrosis	The morphology of tumor cells varies: tubular, papillary, tubular papillary, and so on. The cytoplasm is transparent, deeply stained, bisexual or eosinophilic, and glandular like structures can be seen	AE(+), EMA(+), CK8(+), CK18(+), CEA(+), CK-7(−), PLAP(−)	Ultrasound: irregular cystic masses with septations and unclear boundaries, uneven mixed echogenic lumps	Early occurrence of lymph nodes metastasis and distant metastasis. Regional lymph nodes are mainly retroperitoneal and pelvic lymph nodes, while distant metastasis is mainly in lung, bone, and abdominal organs	Radical resection of the affected epididymis and testicles, assisted with retroperitoneal and pelvic lymph nodes dissection. Radiotherapy and chemotherapy may have limited value
Rhabdomyosarcoma [15–17]	Striated muscle tissues or primitive mesenchymal tissues that differentiates into striated muscle	Gray–white, hard texture, unclear boundaries, may not have bleeding or necrotic areas	The tumor tissues are relatively loose and contain a small amount of mucoid matrix. Tumor cells are circular in shape, with small and deeply stained nuclei and obvious nuclear abnormalities	Myoglobin(+), desmin(+), Ki-67(+), SMA(−), S-100(−), CD34(−), CK(−)	Ultrasound: uneven enlargement of the epididymis, with rich internal blood flow signals and slightly strong swirling echoes MRI: uneven signals inside the nodule, with gradual enhancement on enhanced scans, mainly with edge enhancement. Significant diffusion limitations can be seen in DWI images	Early widespread metastasis to nearby lymph nodes and distant organs such as bone, bone marrow, lung, liver, brain, and breast, with approximately 40% being able to metastasize through blood or lymphatic channels	Comprehensive treatment of radical surgery combined with chemotherapy and radiotherapy Early chemotherapy is sensitive, and the combination of multiple anticancer drugs before surgery has an ideal effect Not sensitive to radiation therapy
Leiomyosarcoma [15]	Smooth muscular tissues	Gray or grayish red, resembling fish flesh, often with bleeding and necrosis, with a hard texture and unclear boundaries to surrounding tissues, often occurring in the tail or head of the epididymis	Tumor cells are elongated spindle shaped, of varying sizes, arranged in parallel or interweaving bundles, with clear cell membranes and eosinophilic staining of the cytoplasm, with visible myofibrils	SMA(+), vimentin(+), CD34(+), S-100(−), EMA(−), CK(−)	Ultrasound: heterogeneous low-echo masses, incomplete capsule, irregular morphology, with abundant blood flow signals, characterized by low-speed and high-resistance blood flow signals MRI: assists in determining regional lymph node metastasis and local involvement	Metastasis occurs less frequently, mainly through blood to distant organs such as the liver and kidney. Lymph node metastasis is rare, but more than 40% of tumors are prone to local recurrence	Radical resection of tumor tissues, generally not requiring lymph node dissection
Fibrosarcoma [7, 18]	Fibroblasts	Purple–red, spherical or lobulated tumor, often with ulceration and bleeding, with a hard texture	Cell nucleus of the tumor is significantly heterotypic, with obvious eosinophilic nucleoli visible. Mitosis is rare, and blood vessels are usually sparse	Vimentin(+), SMA(+), MUC4(+), CD34(+), S-100(−), desmin(−), CK(−), CD68(−), FXIII(−)	Ultrasound: low or uneven echogenicity nodules in epididymis, clear boundaries, with rich blood flow signals	Relatively low-grade malignant sarcoma with a high local recurrence rate; less distant metastasis occurs	Radical resection of tumors, and retroperitoneal lymph node dissection is not recommended for those with negative CT examination results. Postoperative adjuvant radiotherapy and chemotherapy can reduce the probability of local recurrence and metastasis
Myoepithelial carcinoma [19]	Myoepithelial tissues	Gray–white, partially light yellow, slightly tough in texture, with intact capsule, mostly in the form of a single leaf	Tumor cells have diverse forms, with significantly abnormal nuclei and nucleoli. The mitotic figures are easily visible, and pathological mitotic figures can be seen. The cells density is uneven, and the stroma is rich, showing transparent deformation or mucoid degeneration or lacking stromal components	Vimentin(+), SMA(+), p63(+), S-100(+), CK(+), HMB-45(−), MyoD1(−), desmin(−)	Ultrasound: homogeneous weak echogenic masses with irregular shapes, with small areas of no echo visible around them, and linear blood flow signals within the masses MRI: T1W medium signal, T2W slightly high signal, DWI high signal, significantly reduced apparent diffusion coefficient (ADC) value, and progressive nonuniform enhancement on enhanced scans	About one-third of patients experience metastasis and disease progression, including regional lymph nodes metastasis and distant metastasis, with a high local recurrence rate	Radical resection of tumors, supplemented with appropriate radiotherapy and chemotherapy according to specific circumstances
Malignant triton tumor [20, 21]	Neural tissues and skeletal muscle tissues	Gray–white, resembling fish flesh, homogeneous, with a smooth surface and some areas with envelopes	The tumor cells are mainly spindle shaped, arranged in a woven or swirling shape, with rod-shaped or comma shaped nuclei. The chromatin is loose, and there are clear mitotic appearances. The spindle-shaped tumor cells are scattered with abundant cytoplasm and strongly eosinophilic cells, which are circular or polygonal, resembling myoblasts	Vimentin(+), NSE(+), S-100(+), NF(+), CD68(+), CD70(+), desmin(+), myoglobin(+)	Ultrasound: heterogeneous echogenic masses are common, which can appear in a fused shape, with unclear boundaries and irregular shapes, with abundant blood flow signals visible MRI: clumps with long T1 and T2 signals, lobulated edges, high DWI signals, and reduced ADC signals	It is highly malignant and prone to recurrence and metastasis. The recurrence and metastasis rates are both close to 50%, and the median time for recurrence or progression is 6 months	Radical surgical resection is the main treatment method currently, and postoperative radiotherapy can be actively supplemented. Systematic chemotherapy can alleviate advanced or metastatic diseases, but it is generally considered to be insensitive to chemotherapy
Malignant mesothelioma [16, 22]	Mesothelial cells	Gray–red and gray–white solid masses, resembling fish flesh, with a soft texture, often accompanied by multiple focal bleeding and necrosis points, with visible cystic cavities locally	The tumor cells are mostly distributed in the form of sheets and strips in the background of fibroblasts and collagen cells. The spindle-shaped tumor cells have obvious nucleoli, and mitotic figures are easy to see	CK(+), vimentin(+), MC(+), CR(+), SMA(+), actin(+), NSE(+), CD31(+), CD34(+), S-100(−), Ki-67(+), inhibin(−)	Ultrasound: often shows solid slightly high echogenic nodules in the epididymis, with uneven internal echoes and scattered punctate strong echoes. There are few blood flow signals visible MRI: often indicates equal T1W signals, slightly higher T2W signals, and DWI high signals	More than 52% of patients experience local recurrence and metastasis. Retroperitoneal lymph nodes are easily affected	Early radical tumor resection, if there is no metastasis to the inguinal lymph nodes, most scholars believe that preventive lymph node dissection is not necessary; not sensitive to radiotherapy and chemotherapy
Malignant lymphoma [15, 23]	Lymphoid hematopoietic tissues	Gray–white, medium hardness, delicate texture, with necrotic areas visible in the center	A large number of lymphocytes infiltrate the interstitium of tumor cells, with varying sizes of nuclei, appearing circular or oval in shape, with obvious atypia, deep chromatin, and little cytoplasm	LCA(+), CD3(+), Oct3/4(+), CD99(+), CD20(−), CD79α(−), AFP(−), CK(−)	Ultrasound: the normal structure of the epididymis disappears or is not clearly displayed, irregular solid masses can be seen locally, with rich internal blood flow signals	Tumors are highly malignant and grow rapidly, almost always involving adjacent tissues, with early distant metastasis occurring	Radical resection of the testis and epididymis, active adjuvant chemotherapy and radiation therapy
Embryonal carcinoma [24]	Germ cells	The surface is gray–white, gray powder, or gray–brown, soft in texture, with a granular texture, unclear boundaries, and often accompanied by local bleeding, necrosis, and cystic changes	Organizational structures are diverse, with common solid, glandular, and papillary structures. Tumor cells have deep dichroism in the cytoplasm, unclear cell membranes, large nuclei, crowded, overlapping nuclei, pleomorphic and vesicular nuclei, and mitotic figures and apoptotic bodies are common	CD30(+), OCT3/4(+), SOX2(+), SALL4(+), AE1/AE3(+), SOX17(−), glypican3(−), c-kit(−), EMA(−), CEA(−), podoplanin(−), vimentin(−)	Ultrasound: significant thickening of the epididymis or solid masses near the testicle with uneven echoes and rich blood flow signals MRI: mixed signals of bleeding and necrosis in masses CT: slight strips of calcification or punctate calcification, with metastatic lymph nodes fused into masses	Highly malignant tumors, which can have widespread metastasis when the sizes are small, can directly invade the spermatic cord, testicle, and the white membrane, and metastasize to distant organs through lymphatic vessels and blood vessels	Surgical radical resection is preferred, and postoperative adjuvant chemotherapy or radiotherapy can be chosen
Other categories [13, 25, 26]	Such as serous carcinoma, mucinous carcinoma, squamous cell carcinoma, myeloid sarcoma, adenosquamous carcinoma, and so on; all lack typical features						

The content mentioned in the above table represents only some of the relatively typical features reported in the current literature and may not be sufficiently comprehensive

*AE* epithelial keratin, *EMA* epithelial membrane antigen, *CK* cytokeratin, *CEA* carcinoembryonic antigen, *PLAP* placental alkaline phosphatase, *myoglobin* myoglobin, *desmin* anti-desmin, *Ki-67* nuclear protein Ki-67, *SMA* smooth muscle actin, *S-100* soluble acidic protein S-100, *CD34* transmembrane glycoprotein 34, *MUC4* gene structure mucin 4, *CD68* glycoprotein 68, *FX III* small molecule single transmembrane protein, *P63* member of tumor suppressor genes, *HMB-45* melanosomes, *MyoD1* muscle regulatory protein, *NSE* neuroenolase, *NF* nuclear factor, *CD70* transmembrane protein member 70, *MC* member of mesothelial markers, *CR* human calcium binding protein, *LCA* lentil agglutinin, *CD3* membrane glycoprotein complex, *Oct3/4* POU structural transcription factor, *CD99* transmembrane glycoprotein 99, *CD20* transmembrane glycoprotein 20, *CD79 α* B-cell antigen receptor complex associated protein a chain, *AFP* alpha fetoprotein, *SOX2* transcription regulatory factor SOX protein family member 2, *SALL4* transcription factor regulating the development of embryonic abdominal motor neurons, *AE1/AE3* broad-spectrum cytokeratin, *SOX17* transcription regulatory factor SOX protein family member 17, *Glycican3* glycosylphosphatidylinositol proteoglycan, *C-kit* member of the tyrosine kinase receptor protein family, *podoplanin* anti-human flatfoot protein

### Differential diagnosis of malignant tumors in the epididymis

Primary epididymal malignant tumors mainly need to be differentiated from diseases such as epididymal cyst, granuloma of the epididymis, chronic epididymitis, epididymal tuberculosis, benign tumors of the epididymis, and secondary malignant tumors of the epididymis according to medical history, physical examinations, preoperative imaging data, laboratory results, needle aspiration cytology or biopsy, and intraoperative and postoperative pathological results.

### Metastasis of malignant tumors in the epididymis

Common metastasis pathways of epididymal malignant tumors: the most common metastasis pathways of epididymal malignant tumors are hematogenous metastasis and lymphatic metastasis. The lymphatic circulation of the epididymis is similar to that of the testis, and the lymphatic vessels of the two coincide with each other. Their collecting lymphatic vessels follow the internal arteries and veins of the spermatic cord, passing through the seminal vesicle, spermatic cord, inguinal segment, and abdominal segment from the beginning and injecting into the corresponding lymph nodes through the retroperitoneal space. The most common sites of lymphatic metastasis are the common iliac lymph nodes and retroperitoneal lymph nodes, and regardless of whether there is local metastasis, metastasis to retroperitoneal lymph nodes may have already occurred. Therefore, some scholars advocate for routine retroperitoneal lymph node dissection after radical epididymectomy for malignant tumors of the epididymis [27]. Other scholars suggest that patients younger than the age of 10 years do not need to undergo retroperitoneal lymph node dissection if preoperative imaging examinations do not indicate enlarged lymph nodes, while patients older than the age of 10 years must undergo retroperitoneal lymph node dissection if preoperative CT suggests possible lymph node metastasis [28]. The most common sites of hematogenous metastasis are the liver and lungs; metastasis to these sites can occur in the early stages of poorly differentiated squamous cell carcinoma or undifferentiated carcinoma, and the literature suggests that these patients often die within 8 months of diagnosis [5].

### Treatment measures

Treatment principles: at present, when considering the possibility of epididymal malignant tumors, we prioritize preoperative comprehensive evaluation and direct surgical exploration when conditions permit, assist with rapid frozen biopsy during surgery, and then formulate follow-up plans on the basis of the results. If the results indicate a benign tumor, the tumor or epididymis should be removed. If a malignant tumor is considered, radical resection of the epididymis tumor through the inguinal approach is performed, including the tumor, epididymis, testicle, spermatic cord, and tunica vaginalis. When the tumor invades the scrotum, unilateral scrotal resection is needed. If lymph node metastasis in the spermatic cord, pelvic cavity, or retroperitoneal area is considered before surgery, active regional lymph node dissection is recommended [6, 29, 30]. Further comprehensive treatment measures will be determined on the basis of pathological results after surgery. Among them, if the pathological results indicate adenocarcinoma, even if there are no obvious signs of lymph node metastasis temporarily, as it is the main mode of metastasis, active performance of retroperitoneal lymph node dissection supplemented by chemotherapy after surgery is still recommended. Sarcomas are mainly treated with a combination of chemotherapy and radiotherapy, while undifferentiated cancers are mainly treated by radiotherapy with adjuvant chemotherapy if necessary [7].

## Conclusion

As oncologists or surgeons, we should carefully consider the possibility of malignant tumors when facing patients with epididymal tumors. We recommend direct surgical exploration and complete resection of the lesion in one stage, but performing needle aspiration cytology examination before resection to confirm diagnosis is also a feasible option [10, 11]. When pathological result indicates primary adenocarcinoma of the epididymis, retroperitoneal lymph node dissection should be actively performed, and systemic treatments such as chemotherapy and immunotherapy may be temporarily avoided under close follow-up [14, 31]. For other pathological types of malignant tumors, comprehensive measures such as radiotherapy and chemotherapy should be actively taken, referring to relevant information [7, 12, 19, 21, 32].

## Data Availability

PubMed was used as a source of information using the search term: epididymal malignant tumor OR epididymis AND malignancy, epididymis AND cancer, epididymis AND tumor.

## Abbreviations

MRI: Magnetic resonance imaging
CT: Computer tomography
PCK: Pan cytokeratin
EMA: Epithelial membrane antigen
CEA: Carcinoembryonic antigen
WT-1: Wilms tumor gene
CK: Cytokine
CR: Calretinin
CD: Cluster of differentiation
SALL4: Spalt-like transcription factor 4
Ki-67: Nuclear protein Ki-67
OCT3/4: POU structural transcription factor
MC: Member of mesothelial markers
CAM: Cell adhesion molecule
ECT: Emission computed tomography
PET: Positron emission tomography
